# Financial incentives to improve adherence to anti-psychotic maintenance medication in non-adherent patients - a cluster randomised controlled trial (FIAT)

**DOI:** 10.1186/1471-244X-9-61

**Published:** 2009-09-28

**Authors:** Stefan Priebe, Alexandra Burton, Deborah Ashby, Richard Ashcroft, Tom Burns, Anthony David, Sandra Eldridge, Mike Firn, Martin Knapp, Rose McCabe

**Affiliations:** 1 Unit for Social and Community Psychiatry, Barts and the London School of Medicine and Dentistry, Queen Mary University of London, Newham Centre for Mental Health, London, E13 8SP, UK; 2 Division of Epidemiology, Public Health and Primary Care, Department of Epidemiology and Public Health, Imperial College London, Norfolk Place, St Mary's Campus, London, W2 1PG, UK; 3 School of Law, Queen Mary University of London, Mile End, London, E1 4NS, UK; 4 Department of Psychiatry, University of Oxford, University Department of Psychiatry, Warneford Hospital, Headington, Oxford, OX3 7JX, UK; 5 Section of Cognitive Neuropsychiatry, Institute of Psychiatry, King's College London, Denmark Hill, London, SE5 8AF, UK; 6 Centre for Health Sciences, Barts and the London School of Medicine and Dentistry, Queen Mary University of London, Abernethy Building, 2 Newark Street, London, E1 2AT, UK; 7 South West London & St George's Mental Health NHS Trust, Springfield University Hospital, 61 Glenburnie Road London, SW17 7DJ, UK; 8 Personal Social Services Research Unit, London School of Economics and Political Science, Houghton Street, London, WC2A 2AE, UK

## Abstract

**Background:**

Various interventions have been tested to achieve adherence to anti-psychotic maintenance medication in non-adherent patients with psychotic disorders, and there is no consistent evidence for the effectiveness of any established intervention. The effectiveness of financial incentives in improving adherence to a range of treatments has been demonstrated; no randomised controlled trial however has tested the use of financial incentives to achieve medication adherence for patients with psychotic disorders living in the community.

**Methods/Design:**

In a cluster randomised controlled trial, 34 mental health teams caring for difficult to engage patients in the community will be randomly allocated to either the intervention group, where patients will be offered a financial incentive for each anti-psychotic depot medication they receive over a 12 month period, or the control group, where all patients will receive treatment as usual. We will recruit 136 patients with psychotic disorders who use these services and who have problems adhering to antipsychotic depot medication, although all conventional methods to achieve adherence have been tried. The primary outcome will be adherence levels, and secondary outcomes are global clinical improvement, number of voluntary and involuntary hospital admissions, number of attempted and completed suicides, incidents of physical violence, number of police arrests, number of days spent in work/training/education, subjective quality of life and satisfaction with medication. We will also establish the cost effectiveness of offering financial incentives.

**Discussion:**

The study aims to provide new evidence on the effectiveness and cost effectiveness of offering financial incentives to patients with psychotic disorders to adhere to antipsychotic maintenance medication. If financial incentives improve adherence and lead to better health and social outcomes, they may be recommended as one option to improve the treatment of non-adherent patients with psychotic disorders.

**Trial Registration:**

Current controlled trials ISRCTN77769281.

## Background

Various clinical interventions have been tested to achieve adherence in non-adherent patients with psychotic disorders, including compliance therapy, psychotherapy, family education, telephone prompting and psycho education. A review focusing on studies involving patients with chronic health problems [1] and a meta-analysis of studies to enhance adherence in psychiatric patients [2] found a modest effect of some interventions (effect size of .36 in psychiatric patients). Yet, there is no consistent evidence for any intervention to significantly improve medication adherence in non-adherent community patients with psychotic disorders.

Guiffrida and Togerson, 1997 [3] conducted a systematic review on financial incentives to increase adherence to health care treatments. They identified 11 randomised controlled trials, all from the USA. In 10 studies financial incentives enhanced adherence to anti-tuberculosis drugs, dental care, a weight reduction programme, substance dependency treatment, and anti hypertensive medication with odds ratios of up to 7 for anti-tuberculosis treatment. Only one study in the review addressed a mental health issue, i.e. adherence to cocaine dependency treatment. One non-randomised study of patients with dual diagnosis found that modest rewards enhanced attendance to the programme [4].

Since there is no published review specifically on studies in patients with mental health problems, we conducted our own review. The following databases were searched for studies: AMED, EBM, EMBASE, MEDLINE and PsycINFO. The following keywords were combined simultaneously to identify studies: medication, therapy, appointment, compliance, adherence, mental health, mental illness and psychiatr, with the terms incentive, compliance, money, payment, contingency management, voucher and material. We found 13 USA based studies [4-16] where incentives have been used to encourage adherence to treatment in people with mental health problems, 10 of which included people with substance misuse and mental health problems. One study was carried out in the UK [17]. Treatment included attendance of therapeutic sessions and out-patient clinics, and abstinence from smoking or substance abuse. Incentives offered were in the form of a direct payment of vouchers, money or tokens. Nine out of the fourteen studies were within-subjects designs and four studies were controlled trials. Two controlled trials examined the effect of offering incentives to promote abstinence from substances, one studied active involvement in inpatient group meetings and one combined attendance at compensated work therapy and abstinence from substances. In all of the studies, the individuals' adherence/abstinence was significantly improved when incentives were offered. In half of the studies, the improvement in outcome was maintained even when the incentive had been taken away. None of these studies referred to any problems or concerns raised in offering incentives. Yet, we did not find a single controlled study testing financial incentives to improve medication adherence in patients with mental health disorders.

A recent publication from the UK reported the use of financial incentives in non-adherent patients in Assertive Outreach Teams (AOTs) [17]. Four out of 5 patients who were offered the scheme accepted. All had improved adherence to medication and three remained without hospital admissions throughout the observation period although they had been frequently admitted before the scheme. No wider research has so far been published.

The use of financial incentives to increase adherence to anti-psychotic medication also raises ethical concerns as shown in a survey of AOT managers in England [18]. A recent study (Priebe et al, in preparation) explored the views and attitudes of different stakeholder groups related to the use of financial incentives in mental health care. Practically all stakeholder groups identified the issue of effectiveness as critical for their view of the intervention and asked for systematic research to establish its effectiveness.

A clinical trial on the effectiveness of financial incentives will inform the ethical debate on the principle of providing such an intervention [18]. One of four categories for judging the ethical dimension of medical interventions is their beneficence [19]. Beneficence is closely linked to effectiveness, and identifying effectiveness requires a randomised controlled trial. Although there may be various indications that financial incentives are likely to increase adherence to anti-psychotic maintenance medication in previously non-adherent patients, the effectiveness and cost-effectiveness of the intervention has never been established in a randomised design, and a randomised controlled trial is required before the wider use of the intervention should be recommended.

## Methods

### Design

In a cluster randomised controlled trial, community teams caring for patients with psychotic disorders in the community, currently AOTs, will be randomly allocated to the intervention group or control condition. The allocation of teams, and not individual patients, will prevent contamination of practice within teams and facilitate the assessment of overall experiences in teams with the practice. It will also make it possible for teams in the experimental group to offer financial incentives to further patients outside the study, without compromising the study design. Teams might consider this to avoid a sense of unfairness among patients cared for by the same team or simply to have more patients benefiting from the intervention. Outcomes will be analysed on the level of individual patients. The effect of clustering of patients within teams will be controlled for in a mixed effects model. The trial will not be 'blind', as masking of patients and clinicians is impossible. Yet, the primary outcome criterion (percentage of injections taken) and secondary outcomes, with the exceptions of global clinical improvement which is rated by clinicians and subjective quality of life and treatment satisfaction which is rated by patients, can be obtained objectively and are taken from the medical records, and should therefore not be influenced by lack of masking.

AOTs will first be approached and informed about the study through the National Forum for Assertive Outreach and local collaborators at study centres in London, Oxford and Liverpool. We will approach around 100 AOTs that are based within reasonable distance of the study sites so that regular travelling to the teams is realistic. AOTs will receive information about the study on regional and national meetings of AOTs and material circulated through email. This will be followed up by direct telephone calls of the director of the National Forum for Assertive Outreach and other members of the research team including the research assistants. Although we expect that a number of teams will object to either the practice of offering financial incentives or being randomly allocated within a research design or both, informal consultations showed that we can expect more than 36 teams to volunteer for participation in the study. To include an AOT in the pool of eligible teams we will ask for preliminary informed consent by the team manager. AOTs already practising a financial incentive scheme will be excluded. Yet, a survey conducted in 2006 [18] identified only one AOT in England using financial incentives at the time, and this number is unlikely to have increased substantially since. We will then randomly select 36 teams out of the pool of volunteering teams, allowing for two teams to drop out in the further procedure before the trial begins.

All recruited teams will be visited by a member of the research team to explain the nature of the intervention and the study. Clinicians and managers in all teams will receive a structured presentation addressing the research background, the design of the trial, and ethical as well as practical issues of implementation. Written informed consent to participate will then be obtained from team managers and psychiatrist consultants.

The next step will be to identify patients in each team fulfilling the inclusion criteria. We expect the number of patients in most teams to vary between 5 and 8. We expect to recruit 4 patients per team and will randomly select patients if required. These patients will be informed about the study by a clinician and then approached by a researcher for written informed consent for their data to be used in research and for participating in a trial, in which patients in some but not all teams are offered financial incentives to improve medication adherence. If patients cannot be contacted initially or do not provide written informed consent, further patients fulfilling the inclusion criteria will be recruited from the participating teams until the total sample size is reached. Selecting and recruiting patients before randomisation is essential to avoid a possible bias in the selection and recruitment procedures based on awareness of whether patients will be in the experimental or control group. After this one-off contact between the patient and a research assistant, there will be no requirement for further contacts between research assistants and patients in either group. Following the initial interview, patients are not required to participate in any research interviews or assessments at any point of time. This simple and non-intrusive procedure is meant to minimise the number of non-consenting patients (which always is a problem with research in challenging patients in AOTs) and avoid a selection bias as far as possible. Only if patients volunteer to be contacted at the end of the trial again, a researcher will attempt such contact (possibly via telephone) to ask 11 questions on patient reported outcomes.

After the recruitment of patients, 34 AOTs will be randomly allocated to the intervention or control condition stratifying for the type of catchment area (i.e. inner city, suburban or rural).

### Planned interventions

Patients in the AOTs that have been allocated to the intervention will be offered a financial incentive for each depot injection of anti-psychotic medication for a 12 month period. Patients will receive £15 for one injection with the total sum not exceeding £60 for a four-week period (the maximum number of injections is 4 per month). The administering clinician will give the money in cash directly after the injection. Patients will sign a receipt. There are several reasons to set a standard sum of £15 for each depot injection:

• A fixed sum per injection simplifies the practice and makes it transparent for all clinicians and patients involved.

• The sum of £15 is in line with the successful open pilot study in East London.

• The sum is below the limit of £20 per week which would interfere with patients' disability benefits. Most patients eligible for the study receive Disability Living Allowance, Income Support with Disability Premium, or Incapacity Benefit. In all of these cases, patients are not entitled to have a separate income of more than £20 (including therapeutic earnings and income through research participation) without having their benefits reduced.

• £15 per injection is intended to be an incentive helping persuade otherwise ambivalent patients. Yet, it is important to limit the total sum to a maximum of £60 per four weeks so that patients do not become financially dependent on the additional income. The money is intended to provide an incentive, but not lead to financial dependence on the scheme.

Otherwise all patients will receive treatment as usual. The type, frequency and dosage medication and all other interventions will not be affected by participation in the study.

Members of the research team will attend meetings of each AOT in the intervention group and discuss again the practice of offering financial incentives and the nature of the study. Following that there will be a brief training programme on the exact procedure. The procedure of the intervention will also be outlined in a written manual. All teams will then be regularly visited by the research assistants and, if required, also by members of the team of applicants. A discussion of the practice at a team meeting will be repeated after 6 months of the intervention period.

### Inclusion criteria

The only inclusion criterion for teams is that they care for patients with psychotic disorders who have problems adhering to antipsychotic maintenance medication. These are currently dedicated AOTs with a corresponding policy. The only exclusion criteria are lack of willingness to participate and an already existing practice of offering financial incentives to patients with problematic medication adherence.

For patients in the AOTs there are the following inclusion criteria:

• being cared for in the AOT for at least 4 months,

• between 18 and 65 years of age,

• capacity to give informed consent to participate in the study and actual written informed consent,

• an established diagnosis of schizophrenia, schizo-affective psychosis, or bipolar illness according to ICD-10,

• being prescribed depot injections of anti-psychotic medication,

• poor adherence to anti-psychotic medication, i.e. missed 50% or more of prescribed depot injections over the last 4 months (so that the percentage of taken depots is based on a minimum of 4 prescribed depots), and

• failure of all other methods available to the team to ensure adherence to medication.

### Exclusion criteria

Exclusion criteria are:

• learning difficulty

• poor command of English so that clinical communication and discussion of agreements is impaired

### Outcome measures

The primary outcome is adherence to anti-psychotic maintenance medication during the 12 month trial period. Adherence will be measured, objectively, as the percentage of prescribed depot injections actually taken. As the primary outcome, the percentage will be used as a continuous variable. However, we will also analyse the percentage in a dichotomised way, comparing the ratio of patients with 'good' adherence (i.e. =80% of prescribed depots taken [20]) in the two conditions.

Further secondary outcomes are:

a) The time 'slippage' of taking depots, defined as the percentage of the prescribed time interval that has expired before the depot is taken;

b) Clinical improvement as assessed on the Clinical Global Impression Scale (CGI) [21] by the treating consultant psychiatrist at the end of the 12 month period;

b) Number of involuntary and voluntary hospital admissions during the trial period;

c) Costs of care: data on the use and frequency of use of inpatient care, outpatient care (including home visits, home treatment), and other health services during the 12 month treatment period will be obtained from case notes and electronic administrative data bases. Costs for the intervention will be estimated for each participating team from information provided by staff. Established national unit costs will be used to estimate direct health care.

d) The number of attempted and completed suicides, incidences of physical violence, police arrests and days spent at work/training/education will also be recorded over the 12 month trial period.

e) Subjective quality of life and satisfaction with medication which will be assessed at the beginning and end of the intervention period using the 11 item scale established in the DIALOG trial [22]. The scale contains 11 items asking patients to rate their satisfaction with 8 life domains and 3 treatment aspects, one of which is medication, on a scale ranging from 1 (lowest satisfaction) to 7 (highest satisfaction).

f) Continuation with financial incentives (in intervention group only) and adherence during a 6 month follow up period will be taken from the medical records.

g) Teams in the intervention group will be asked after 6 months, 12 months and 18 months about all aspects of experiences with the scheme including whether patients on the scheme asked for an increase of the incentive, and whether other patients with hitherto good adherence also asked for financial incentives and/or became poorly adherent in order to be eligible for the incentives. This will be done using open questions with a written documentation of the answers.

Simple measures of subjective quality of life and satisfaction with medication are the only patient reported outcome criteria used in the study. They have been included to obtain a subjective outcome that reflects the user perspective. However, this will be an element that patients can participate in or not. If they do not consent to be contacted for completing the scale at the end of the intervention period, they will still participate in the trial, and there are no mandatory patient rated or interview based criteria. The patients to be recruited for the trial have been 'difficult to engage' in care, and many may refuse participating because they do not want to be interviewed by a researcher or complete questionnaires. This would result in difficulties to recruit and - more importantly - a significant selection bias.

### Risks and anticipated benefits for trial participants and society including how benefits justify risks

There are potential risks linked to offering financial incentives for patients in the intervention group. These include that patients a) become financially dependent on the incentive, b) demand more money over time, c) will not want to terminate the scheme although they might be prepared to adhere to medication even without the incentive, and d) spend the additional income on illegal drugs. Also, other patients who have been adherent so far might ask to be offered financial incentives as well and/or decrease their adherence to become eligible. Based on 5 years experience with the intervention in the AOT in the East London Borough of Newham, one can expect most of these risks to be limited. No patient with good adherence has ever asked to receive financial incentives as well (and to our knowledge none has ever become poorly adherent in order to be eligible for the scheme). One patient receiving the intervention has once asked for the money to be increased which was declined without any negative consequences. The financial dependence is difficult to judge, but the maximum overall amount of £60 per 4 week period is rather small to induce dependence. We cannot guarantee whether patients spend the additional income on illegal drugs, but all patients have civil rights and the capacity to decide on what they want to spend their money and, on a practical level, the amount of incentives is not sufficient to fund a significant use of illegal drugs.

The anticipated benefits for the patient include a much better quality of life with reduced distress, lower suicide risk, fewer problems with the justice system, lower rate of compulsory treatment and less time spent in psychiatric in-patient units. Some patients may see the benefit of the medication, change their attitude towards it and later take it without financial incentives [23].

The potential benefits to society include a reduced risk of patients to harm others and much lower costs in terms of input of health services and other services in the society including the police and the justice system.

For patients in the control group there are no discernible risks or benefits. They will only be asked whether they consent to their data being used for research and would consider in principle an offer of financial incentives to take their medication. Their care will not be altered at all. For patients in either group there are a maximum of eleven satisfaction ratings on one scale, but no potentially distressing interviews or assessments. In the intervention group, patients get offered financial incentives, but can refuse further financial incentives and medication itself at any point of time.

### Trial Steering Committee and Data Monitoring Committee

A trial steering committee (TSC) will be established with an independent chair, a user representative, and at least two further independent experts.

Although the amount of data collection is limited in the trial, we will also establish a Data Monitoring and Ethics Committee (DMEC) because of the ethically sensitive nature of the intervention. The DMEC will be independent of the applicants and report to the TSC. It is suggested to have a joint TSC/DMEC meeting at the beginning of the study, and subsequently arrange DMEC meetings before the TSC meetings. The meetings of both groups will be scheduled for times immediately following the expected delivery of major milestones, i.e. in month 6, 12 and 25 of the study

### Ethical approval

The study has been approved by Ealing and West London Research Ethics Committee (REC reference number: 09/H0710/35). All data will be anonymised and stored securely in line with the Data Protection Act. No published data will contain patient identifiable information.

### Statistical analysis

For analysis, we will use generalized linear models as appropriate to the outcome, with random effects for groups, and sensitivity analyses to explore the impact for missing data. A detailed Statistical Analysis Plan will be agreed by the TSC prior to analysis of un-blinded data.

### Economic analysis

A cost-effectiveness analysis will be conducted from an NHS perspective, using data on health service use, national unit cost figures and the main outcomes in turn (adherence, time 'slippage' of taking depots, CGI). Incremental cost-effectiveness ratios and cost-effectiveness acceptability curves will be estimated and employed as necessary, generated from the net benefit approach and using bootstrap regression for a range of values of willingness to pay for incremental outcome changes. Sensitivity analyses will examine the impact of altering key assumptions and parameter values. It is usual in any trial to find differences in service access, treatment adherence, baseline characteristics, changes in outcome dimensions over time, cost and cost-effectiveness. In the present study, these variations would be of particular interest, and we therefore plan to analyse patterns within the samples in order to examine whether there are identifiable patterns of inequity with respect to need, socioeconomic group, and key demographic characteristics. The concentration index approach, now quite widely used in health economics for example, offers a robust and informative methodology [24].

### Proposed sample size

**Figure 1:**
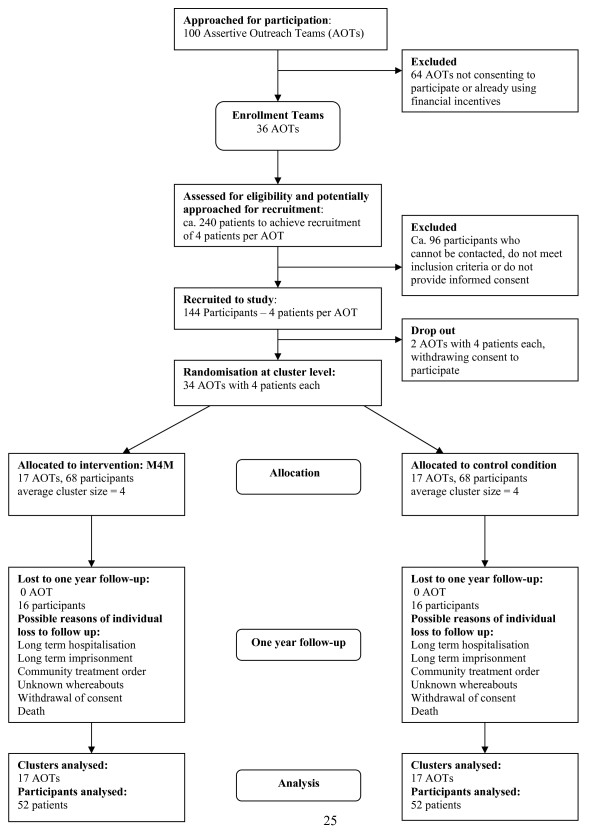
**This figure displays the CONSORT Flow Diagram**.

We will recruit 34 AOTs in England (initially 36 to allow for two teams to drop out between recruitment and beginning of trial), and 4 patients within each team. Seventeen teams each will be randomly allocated to the experimental group and the control intervention, i.e. 'treatment as usual'. We aim to have 68 patients in each arm of the trial, allowing for one patient per team to be lost between recruitment and one year follow up. This estimate of a loss of one patient per team may be rather pessimistic, but enables us to have a minimum of 52 patients per arm (assuming that in at least one team per arm there will be no loss to follow up) included in the intention-to-treat analysis. Dropping out of the study and the intention-to-treat analysis will occur only because of a) death, b) long-term imprisonment, c) long-term hospitalisation, d) unknown whereabouts with no chance to obtain outcome data, or e) withdrawal of consent for the data to be used for research. Patients in the intervention group may discontinue with the intervention within the one year study period, because their clinicians think that maintenance medication is not appropriate anymore or patients themselves decide to come off the scheme. Such patients will still be included in the intention to treat analysis, and discontinuing with the scheme will not compromise the availability of outcome data. Refer to Figure 1. CONSORT Flow Diagram for details.

According to the definition of good adherence as taking at least 80% of prescribed medication, the study is powered to detect a difference in adherence from 25% in the TAU arm to 65% in the experimental arm with 90% power for 5% significance. To convert this to a continuous measure requires an estimate of the standard deviation of the percentage of medication taken: Remington et al 2007 [20] estimate this as 31%. This estimate may appear high, which makes our power calculation rather conservative. Assuming the 31% standard deviation pertains to both arms, the original assumptions are then equivalent to assumed means of 60% of prescribed medicine on TAU, and 92% on treatment. In fact, the mean in Remington et al [20] was 66%, so the revised sample size calculations on the continuous measure are powered for a more modest increase from 65% to 85% (an absolute difference of 20%). This would require 47 per group in an individually randomised study. This then has to be inflated to allow for clustering. Assuming an ICC of 0.05 and an average of 3 patients per team gives an inflation factor of 1.1 [25] or 52 per group. We will therefore aim to have one year follow up data from at least 3 patients each from 17 teams per arm (and for 4 patients in one team in each arm). To allow for potential dropout we will actually recruit 4 per team. We do not propose a correction for variable group size. The numbers recruited per team are under our control and the loss to follow-up rate is likely to be low with small differences between teams. Thus, the coefficient of variation of the group sizes is unlikely to exceed 23% [26].

## Discussion

The trial aims to establish the effectiveness and cost effectiveness of offering financial incentives to improve adherence to antipsychotic maintenance medication. The target group are patients with psychotic disorders who do not adhere to medication, although they are likely to benefit from it. The primary outcome is adherence to medication. We assume that an improved adherence to medication will be associated with significant health and social gains for the patients concerned. However, the aim of this trial is only to test an intervention to improve adherence and not whether antipsychotic maintenance medication is indeed effective or not. We therefore decided to use health and social outcomes only as secondary criteria, although improving them is the ultimate objective of the whole intervention.

It would also have been desirable to have patient reported measures, e.g. on their attitude to treatment in general and to medication in particular, as a central outcome. However, the target group of this study are very difficult to engage in care and often even more difficult to engage in research trials. Requiring patients to attend interviews or fill in questionnaires may have limited recruitment and lead to substantial drop out rates. Thus, the ideal research design cannot be implemented because patients are likely not to comply.

We plan to conduct the study with and in AOTs. Yet, given the possible organisational changes in the NHS which can be difficult to anticipate, we may have to deal with teams that are re-configured during the duration of the trial. The research team will try and implement the study protocol despite such changes. We will aim to ensure that the allocation of patients to teams in the experimental arm or control group throughout the study period is not compromised. This may be a challenge since the research team has no managerial or clinical control over the participating clinical teams. We will also aim to assess the general experiences of teams with the practice, e.g. whether other hitherto adherent patients also asked for financial incentives and whether teams continue with offering financial incentives to the study patients after the end of the trial. These general experiences may be highly relevant for a potential wider implementation of the intervention.

If the trial shows that offering financial incentives is effective and cost-effective, it may be recommended as an option in the treatment of patients with psychotic disorders who are non-adherent to medication. The measure is not coercive and requires patients to have full capacity to make the decision to both taking the medication and accepting a financial incentive. There is no reliable data on the exact size of that group of patients. One may estimate that between 1000 and a maximum of 5000 patients in the UK may fall into this category at one point of time. However, the implications of a positive finding may go beyond the UK and also affect treatment of similar patients in other countries.

## List of abbreviations used

AOT: Assertive Outreach Team; CMHT: Community Mental Health Team; DMEC: Data Monitoring and Ethics Committee; FIAT: Financial Incentives (for) Adherence Trial; ICC: Intracluster Correlation Coefficient; NHS: National Health Service; TAU: Treatment As Usual; TSC: Trial Steering Committee.

## Competing interests

SP is principal investigator (and RA and MF are co-investigators) on a qualitative study on ethical concerns related to the use of financial incentives to improve adherence to antipsychotic maintenance medication in patients with poor adherence (funded by the Wellcome Trust. Ref No. 081433/Z/06/Z). SP was also co-author on a case observation study in which five assertive outreach team patients were offered financial incentives to adhere to medication [17].

## Authors' contributions

All authors have read and approved the final manuscript. SP coordinated and wrote first the outline application and then the full application to the HTA (Health Technology Assessment Programme of the National Institute for Health Research). All other named authors contributed to the study design and protocol in the following ways: DA and SE contributed to the trial methodology and statistical analysis plan; RA provided expertise in bioethics; TB, RM, AD and MF contributed to methodological and practical aspects of the design and influenced the plans to implement the design; AB contributed to the development of the protocol and application procedure; MK provided the plans for the economic analysis.

## Pre-publication history

The pre-publication history for this paper can be accessed here:


